# Predicted functional interactome of *Caenorhabditis elegans* and a web tool for the functional interpretation of differentially expressed genes

**DOI:** 10.1186/s13062-020-00271-6

**Published:** 2020-10-19

**Authors:** Peng-Cheng Chen, Li Ruan, Jie Jin, Yu-Tian Tao, Xiao-Bao Ding, Hai-bo Zhang, Wen-Ping Guo, Qiao-lei Yang, Heng Yao, Xin Chen

**Affiliations:** 1 Institute of Pharmaceutical Biotechnology of Zhejiang University School of Medicine and Department of Radiology of the First Affiliated Hospital, Hangzhou, 310058 China; 2 Institute of Big Data and Artificial Intelligence in Medicine, School of Electronics and Information Engineering, Taizhou University, Taizhou, 318000 China; 3 Joint Institute for Genetics and Genome Medicine between Zhejiang University and University of Toronto, Zhejiang University, Hangzhou, 310058 China

**Keywords:** *Caenorhabditis elegans*, Functional interaction, Database, Gene set linkage analysis, Transcriptomic analysis tool

## Abstract

**Background:**

The nematode worm, *Caenorhabditis elegans*, is a saprophytic species that has been emerging as a standard model organism since the early 1960s. This species is useful in numerous fields, including developmental biology, neurobiology, and ageing. A high-quality comprehensive molecular interaction network is needed to facilitate molecular mechanism studies in *C. elegans*.

**Results:**

We present the predicted functional interactome of *Caenorhabditis elegans* (FIC), which integrates functional association data from 10 public databases to infer functional gene interactions on diverse functional perspectives. In this work, FIC includes 108,550 putative functional associations with balanced sensitivity and specificity, which are expected to cover 21.42% of all *C. elegans* protein interactions, and 29.25% of these associations may represent protein interactions. Based on FIC, we developed a gene set linkage analysis (GSLA) web tool to interpret potential functional impacts from a set of differentially expressed genes observed in transcriptome analyses.

**Conclusion:**

We present the predicted *C. elegans* interactome database FIC, which is a high-quality database of predicted functional interactions among genes. The functional interactions in FIC serve as a good reference interactome for GSLA to annotate differentially expressed genes for their potential functional impacts. In a case study, the FIC/GSLA system shows more comprehensive and concise annotations compared to other widely used gene set annotation tools, including PANTHER and DAVID. FIC and its associated GSLA are available at the website http://worm.biomedtzc.cn.

## Background

More than 50 years ago, Sydney Brenner selected *Caenorhabditis elegans* as a genetic model to study developmental biology and neurobiology because of its fully mapped genome and nervous system, rapid life cycle (3 days), and ease of laboratory cultivation [1–4]. The experimental strength and physiological similarities present in *C. elegans* and higher organisms (e.g., humans) have made it an important organism to explore a variety of subjects, including neuron fate specificity [5], axon guidance [6], and differentiation [7].

Recently, the development of omics technologies for *C. elegans* has become indispensable to systems biology, which can help to elucidate the mechanisms governing cellular physiology at the molecular level [8, 9]. Although the variety and complexity of omics data provide a global overview of potential mechanisms of physiological change, it also presents unparalleled challenges to describe the underlying design logic of physiological processes from molecular-level descriptions.

In fact, the existing approaches mostly rely on enrichment analysis to obtain high-level biological insights from the observed set of differentially expressed genes (SDEG). These enrichment-based methods evaluate whether an SDEG is enriched or clustered in a defined biological process. To date, many gene set annotation tools based on enrichment analysis have been developed to analyse SDEG, and they show significantly different expression in two or more physiological statuses. Some tools are widely used, including PANTHER [10], KEGG [11], and DAVID [12].

In reality, the observed SDEG is summarized into established biological concepts by the above strategies, which are successful in many cases. However, in practical use, enrichment-based methods frequently report that no annotation term is enriched or only report conceptually general terms (such as GO: 0005634, nucleus), where no established biological concepts can be used to accurately describe the differentially expressed gene set. Clearly, these results provide little assistance for researchers attempting to elucidate molecular mechanisms.

Alternatively, while there are no established concepts to accurately describe the observed SDEG, we still may utilize established biological concepts to interpret the functional impacts of SDEG. For example, observed SDEG may lead collectively to GO: 0051704 (defence response to fungus), even when the SDEGs themselves are not enriched in these terms (for details, please see the Discussion section). In this study, the gene set linkage analysis (GSLA) tool was developed to describe the potential functional impacts of the observed SDEG, especially in cases in which no established concepts or no suitable concepts can describe these changes. If the SDEG has strong functional associations with genes in an established biological process, the observed SDEG will be expected to interfere with this biological function. This strategy is called GSLA and has been successfully used in human and Arabidopsis transcriptome interpretation [13, 14]. The main contribution to the successful interpretations of functional impacts is the high-quality functional association networks in these two species [13, 15]. Worm researchers have made efforts to construct molecular interaction databases, which include WormBase (2390 proteins, 6343 interactions) [16], WormNet (16,122 proteins, 760,116 interactions) [17], mentha (4774 proteins, 12,136 interactions) [18], MIST (4929 proteins, 626,262 interactions) [19], STRING (12,050 proteins, 3,300,700 interactions) [20], and ComPPI (4958 proteins, 13,659 interactions) [21]. These databases have greatly facilitated *C. elegans* research, but they do not support the gene set linkage analysis (GSLA) algorithm well in our evaluation.

Therefore, in this study, we constructed a high-quality reference functional gene association network for *C. elegans*, which provides an alternative resource for high-reliability functional gene associations to facilitate mechanism exploration. Additionally, this set of functional gene associations supports the GSLA interpretation of the collective functional impacts of SDEGs in worm. The predicted functional interactome of *Caenorhabditis elegans* (FIC) integrates six types of functional association data from 10 public databases with timestamps before 2018. To evaluate the accuracy of inferred functional associations in FIC, we used experimentally confirmed protein-protein interactions recently reported after 2018. The current version of FIC includes 108,550 functional gene associations, which are expected to cover 21.42% of protein-protein interactions in *C. elegans,* and 29.25% of functional gene interactions may represent protein interactions. For users to query functional associations of their genes of interest in *C. elegans*, we provide an FIC web interface that is easy to operate. We also provide a GSLA web tool so that users can interpret the potential functional impacts of the observed set of differently expressed genes. A case study is also provided to illustrate the way to use the FIC/GSLA system.

## Methods

### Evidence data

In this work, we selected six types of evidence that suggest functional associations between genes from 7 public databases to build the interaction prediction model. These data were collected before 2018 and include 18,947 expression profiles (Coxpresdb), 44,300 gene annotations (GOC), 35,195 domain interactions (IDDI and Pfam), 14,457 subcellular gene localizations (Compartments), 14,866 phylogenetic profiles (DIOPT), and inparalogue/orthologue relationships between 5137 worm proteins and proteins from *A. thaliana, D. melanogaster, H. sapiens, M. musculus, R. norvegicus, S. cerevisiae* and *S. pombe* to compute interologues. Thirty-six features belonging to six categories were computed based on these evidence data, each suggesting a certain type of functional association (Supplementary Table 2) [22, 23]. Detailed methods and equations can be found in website help (the indirect evidence section).

**Fig. 1:**
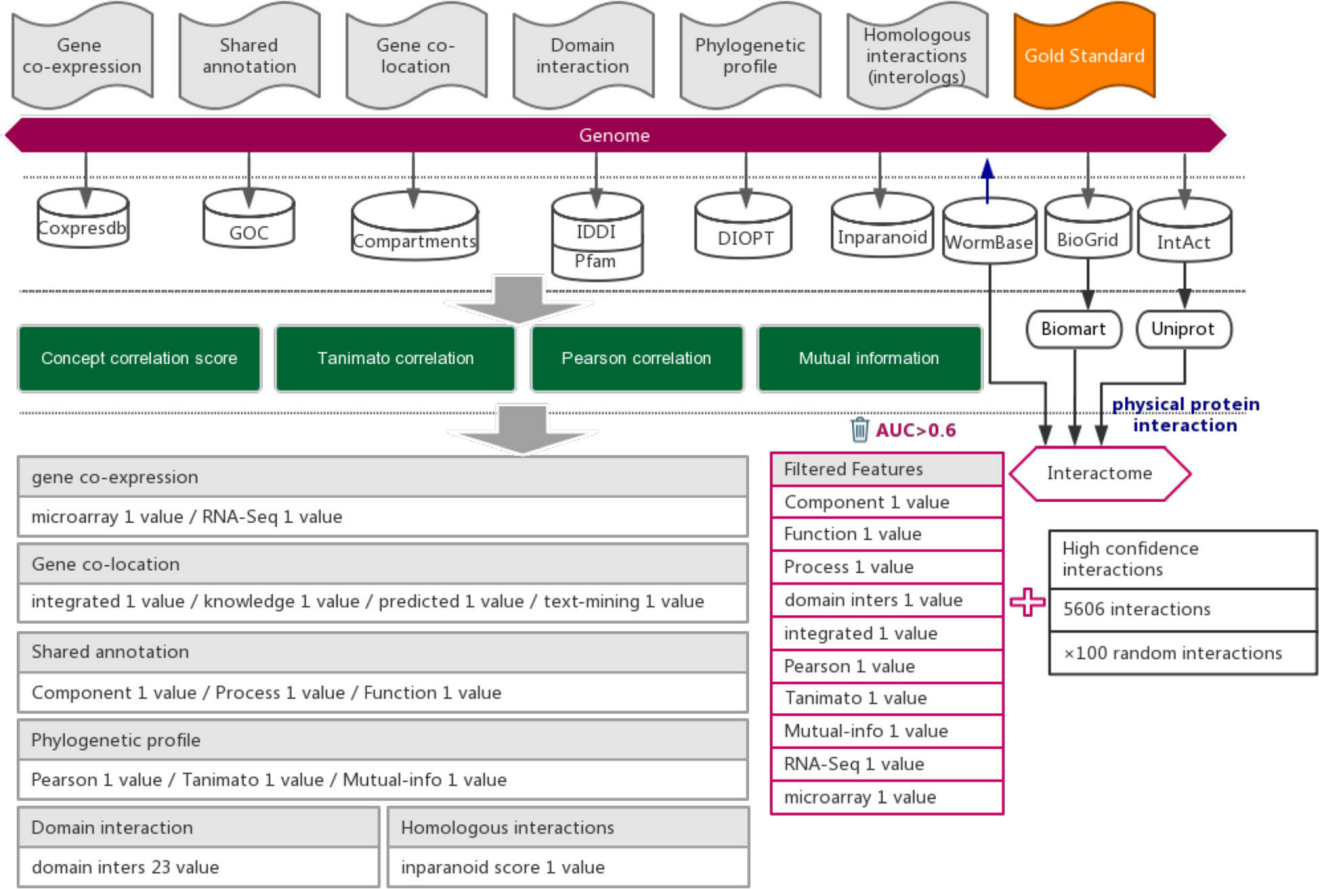
Workflow for inferencing functional interactions between *C. elegans* genes. High-quality experimentally reported protein interactions were integrated from three databases and were used as positive examples. Six types of functional association evidence from 10 databases were collected to infer putative functional interactions. A total of 10 high-quality feature values were selected from 36 feature values that characterize this evidence with different mathematical representations. Random gene pairs after removing positive examples were used as negative examples. The number of negative examples was 100 times that of the positive examples

Protein-protein interactions were considered to be evidence of strong functional associations. In this study, we attempt to predict functional associations that are as strong as protein interactions [13]. We collected a total of 26,367 experimentally reported protein-protein interactions of *C. elegans* from three public databases, including WormBase [16], BioGRID [24], and IntAct [25] (Additional file 1: Table S1). Among 26,367 interactions, we retained 5606 high-quality protein-protein interactions to ensure that these interactions are experimentally confirmed, rather than predicted. The interactions that were reported in less than two independent studies or reported only in high-throughput experiments were removed (Additional file 1: Table S1). To obtain the uniform gene ID for the functional association prediction, UniProt [26] and BioMart [27] software were used to convert different gene IDs to WormBase ID (Fig. 1).

### Computation and assessment of feature values

From six types of functional association evidence, we used 36 mathematical characterizations to compute feature values, which include 3 shared annotation features, 2 co-expression features, 4 subcellular co-localization features, 23 domain interaction features, 3 phylogenetic profile features, and 1 homologous interaction (Additional file 3: Table S2).

To evaluate whether the above feature values can suggest functional associations, the area under the curve (AUC) of the receiver operating characteristic (ROC) test was employed. Each feature value produced a series of sensitivities and specificities by applying different cut-offs when predicting the protein interactions. According to different cut-offs, the ROC curve was plotted with sensitivities and specificities (X-axis, 1-specificity; Y-axis, sensitivity). Assessment of feature values was performed using the training dataset (data before 2018). If the AUC of a feature value was higher than 0.6, a strong functional association was present. A total of 10 features were eventually selected to predict the functional associations (Additional file 4: Table S3 and Additional file 2: Fig. S1).

### Website construction

Our developed FIC database runs on LNMP that integrated the running environment, including Linux, Nginx, MySQL, and PHP. Data storage, maintenance, and operation are supported by the MySQL database. The interaction interface is developed with the Laravel framework based on PHP. The front-end of FIC is a Vue.js-based single page application (SPA). Vue.js is an open-source JavaScript framework for interface creation and a web application framework compatible with SPA. The functional association network is visualized with Cytoscape [28].

### Microarray data analysis

From the GEO database [29, 30], we retrieved microarray data GSE97678 [31]. In the original article, 35 genes were upregulated between 2- and 8-fold on the *E. coli* HT115 diet, while 22 genes were upregulated between 2- and 20-fold in the *E. coli* OP50 diet. Three biological replicates were performed by the authors. These expression profiles were re-analysed based on the online GEO2R tool [32–34] using default parameters. The top transcriptionally changed genes were selected by adj. P. Val (*P*-value after adjustment for multiple testing). The GEO2R tool adjusts the *P*-values to correct for false positive results. We chose the default Benjamini & Hochberg false discovery rate method to adjust multiple tests.

## Results

### Data integration for functional association prediction in *C. elegans*

Six types of evidence for functional association inference between *C. elegans* genes were collected from seven databases, including Coxpresdb [35], Gene Ontology Consortium (GOC) [36], Compartments [37], IDDI [38], Pfam [39], DIOPT [40] and Inparanoid [41] (Fig. 1). Thirty-six feature values from six types of evidence were used to measure the strength of functional associations (Additional file 3: Table S2).

Not all 36 of these features were suitable to separate protein interactions from random gene pairs. To decrease the noise-to-signal ratio in the following functional associations prediction step, we only kept those features that showed a strong correlation to functional associations. The AUC of the receiver operating characteristic (ROC) curve was used to measure the capability of a feature to indicate protein interactions. In this study, we selected ten features with AUC higher than 0.6 for the subsequent inference of functional gene associations (Additional file 4: Table S3 and Additional file 2: Fig. S1).

In addition, protein-protein interactions reported in experimental studies of *C. elegans* were collected from three databases, including WormBase [16], BioGRID [24], and IntAct [25] (Fig. 1 and Additional file 1: Table S1). We removed protein-protein interactions based on the supporting evidence provided in each database (Fig. 1), only retaining the experimentally confirmed high-quality protein-protein interactions, which were used as positive examples in prediction model training (Additional file 1: Table S1).

### Functional gene association prediction

We used the libSVM package to train and predict functional associations [42, 43] (Fig. 1). Specifically, 5606 high-quality experimentally confirmed protein-protein interactions that were published before 2018 were used as positive examples, which represent examples of strong functional associations between *C. elegans* genes. Randomly generated gene pairs that do not overlap with positive examples served as negative examples. We considered that two random genes may have functional associations, although the probability is low. To reduce the false positive rate in the negative examples, we set the positive-to-negative ratio in the training dataset to be 1:100 such that only a notably small fraction of gene pairs has functional associations.

We utilized the soft-margin Gaussian kernel SVM algorithm to train the prediction model. The parameters σ (kernel width) and C (soft margin) targeted an optimal harmonic mean of sensitivity and specificity that were optimized with a 5-fold cross-validation. Based on the optimized σ and C, all training data were used to train the prediction model, which was validated with an external validation dataset consisting of protein-protein interactions that were published after December 31, 2017, and randomly generated negative examples. After validation, this model showed a sensitivity of 21.42% and a specificity of 99.95%. In this study, we also evaluated how well the predicted interaction in WormNet, MIST, and STRING covered these new interactions. The results are shown in Supplementary Table S4.

Applying this model to all gene pairs of *C. elegans* produced 101,727 inferred functional associations. These inferred functional interactions together with the 6823 known protein interactions make the FIC dataset, which consists of 108,550 interactions. A total of 108,550 putative functional interactions were inferred when applying this model to all *C. elegans* gene pairs. Among the protein-protein interactions, we further estimated the proportion covered by the predicted functional interactome using the following equation:

$$ {N}_{interactome}\times Sensitivity+\left({N}_{all- pairs}-{N}_{interactome}\right)\times \left(1- specificity\right)={N}_{\mathrm{predict}} $$

where *N*_*interactome*_ is the expected number of all protein-protein interactions in *C. elegans*, *N*_*all* − *pairs*_ is the number of all gene pairs in *C. elegans*, and *N*_*predict*_ is the number of predicted gene associations. The sensitivity and specificity are the accuracy measures produced when the prediction model was validated with newly published protein interactions. Solving this equation yields an estimated size of 1.42 × 10^5^ for the *C. elegans* protein interactome. Based on the estimated interactome size (1.42 × 10^5^) and the estimated sensitivity (21.42%, the conservative estimation from the training stage sensitivity 21.69% and the evaluation stage sensitivity 21.42%), the predicted interactions in FIC are expected to include 29,755 protein interactions. Therefore, 29.25% of the FIC functional interactions (29,755 out of 101,727) are expected to represent protein interactions.

### Assessment of functional gene association network

We evaluated the quality of the functional gene association interactome FIC by its ability to group functionally associated genes together. A gene’s function prediction accuracy based on its network neighbours may be used to measure the quality of our predicted interactome FIC. To this end, the quality of the inferred functional associations was compared with six other public *C. elegans* interactomes, including WormBase [16], WormNet [17], mentha [18], MIST [19], STRING [20], and ComPPI [21]. In this study, the PATHER term enrichment tool [10] was used to measure the accuracies to predict the new GO biological process annotations on each interactome.

The collection date of data for the inference of FIC gene associations was before 2018 (December 31, 2017). We collected 13,043 genes from GO [44, 45] with new annotations dated up to August 1, 2018. These genes contained a total of 117,848 annotations, 9108 annotations of which were newly added after 2018. We evaluated the prediction performance of gene function based on these genes and their annotations.

The overall prediction accuracy of new annotations across seven interactomes was compared by precision-recall curve. In this instance, precision means whether the annotations reported by PANTHER are consistent with the known annotations (all 117,848 annotations), while recall means the proportion of annotations reported by PANTHER that covered the newly added 9108 annotations. The number of PANTHER reported annotations is correlated with the cut-off on the significant value. Setting a higher cut-off value results in more reported annotations and a higher recall but with a higher false positive rate. In contrast, setting a lower cut-off value results in fewer reported annotations and lower recall but with higher precision. Therefore, the advantage of the precision-recall curve is that it shows the rates of precision and recall on different cut-offs. Independent of the selection cut-offs, the precision-recall curve can provide a more comprehensive view of the quality of the interactome.

**Fig. 2:**
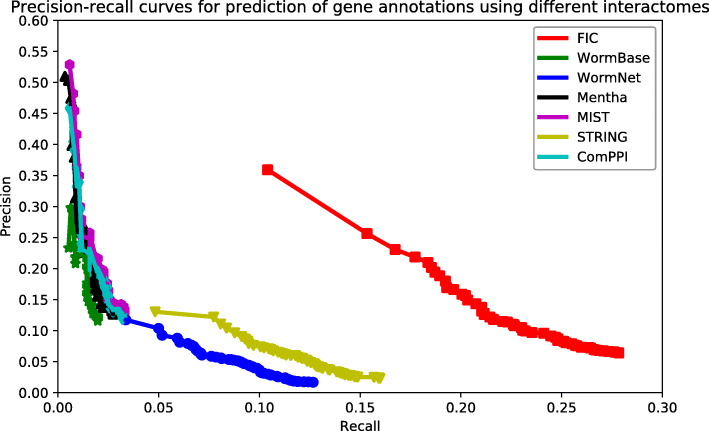
Assessment of the capabilities of seven interactomes to group functionally associated genes together. The precision-recall curves of gene function prediction using different interactomes are illustrated. Precision estimates the proportion of correct annotations identified by an interactome. Recall estimates the proportion of new annotations that is identified by an interactome

Figure 2 shows that the curve of our predicted FIC interactome is above six other interactomes, indicating its superior quality in grouping functionally associated genes together. Compared to others, only the curve of FIC reaches the high-recall region, and it still maintains the highest precision. Although WormBase, mentha, MIST, and ComPPI have similar high-precision regions, none of these curves reached high-recall regions. Alternatively, STRING and WormNet reached the high-recall region, but their precision did not increase considerably and always stayed in the low-recall region. This observation suggested that a high proportion of STRING and WormNet interactions were weak functional gene associations, which may raise the false positive rates during function prediction. In general, FIC showed balanced coverage and accuracy, and its quality exceeds those of other compared interactomes.

### Web interface of FIC/GSLA

**Fig. 3:**
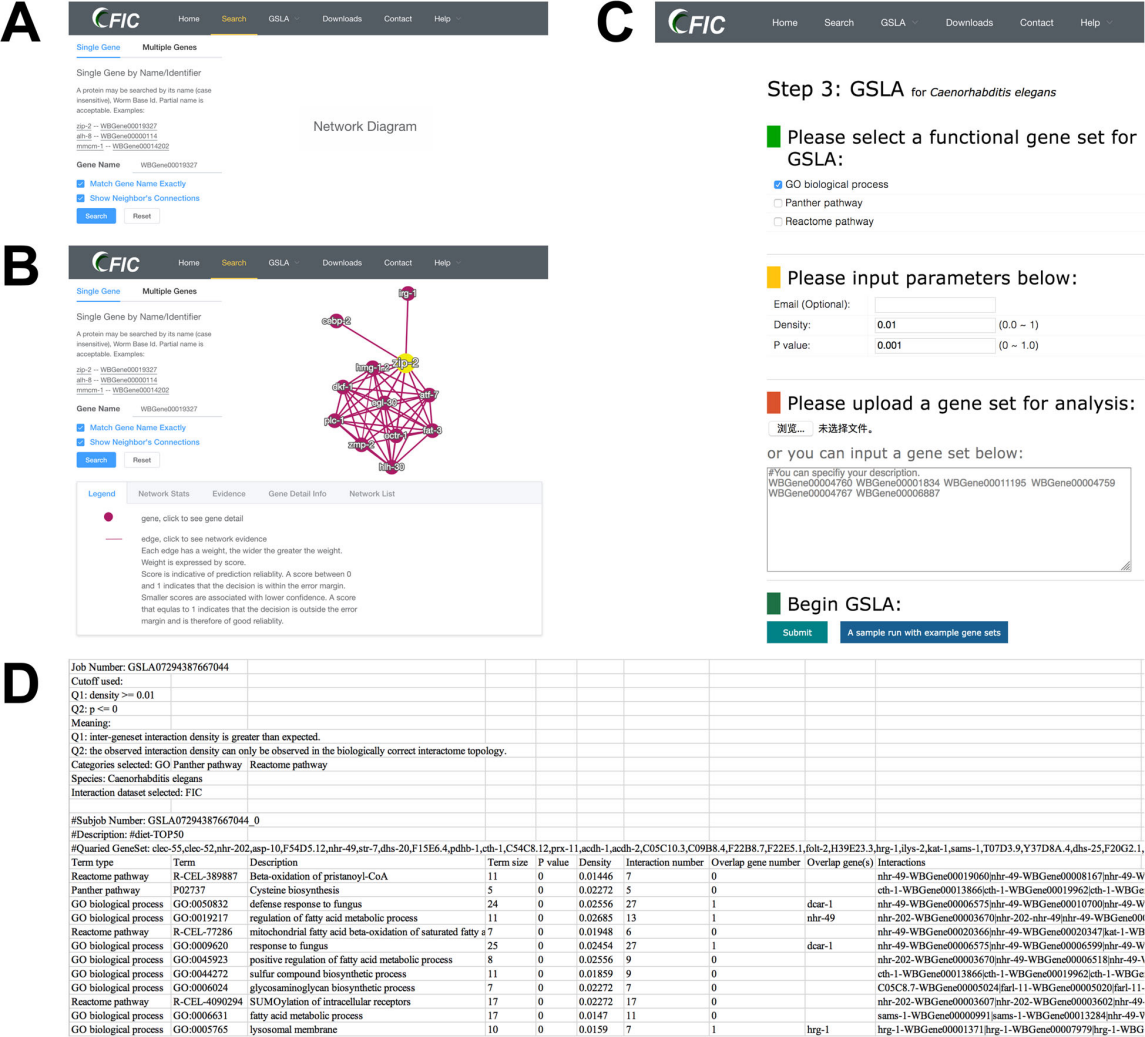
Interface of FIC and GSLA. **a** Two search options in FIC. **b** Search result page. A right click on the edge and node will show interaction details. **c** Interface of GSLA. **d** Results of a GSLA analysis job

We developed a user-friendly interface for FIC, which provides two search modes, a single gene search mode and a multiple gene search mode (Fig. 3a). Both gene name and WormBase ID are acceptable means of searching for a gene. The single gene search option reports putative functional gene-associated interactions involving the query gene, while the multiple search option reports the functional associations between query genes. The functionally associated interactions reported by FIC are provided in a tabular form (Fig. 3b). A graphical view of these reported interactions is also provided at the right side of the query interface. Users can check for feature values for the interaction prediction in our model if they click the edges in the graphical view of their functional associations. Moreover, users can click the nodes in the graphical view to obtain gene information that provides more detailed information about a gene. A full dump of the FIC database is available for download. We also provide a help section on the FIC/GSLA website with more details for users.

**Fig. 4:**
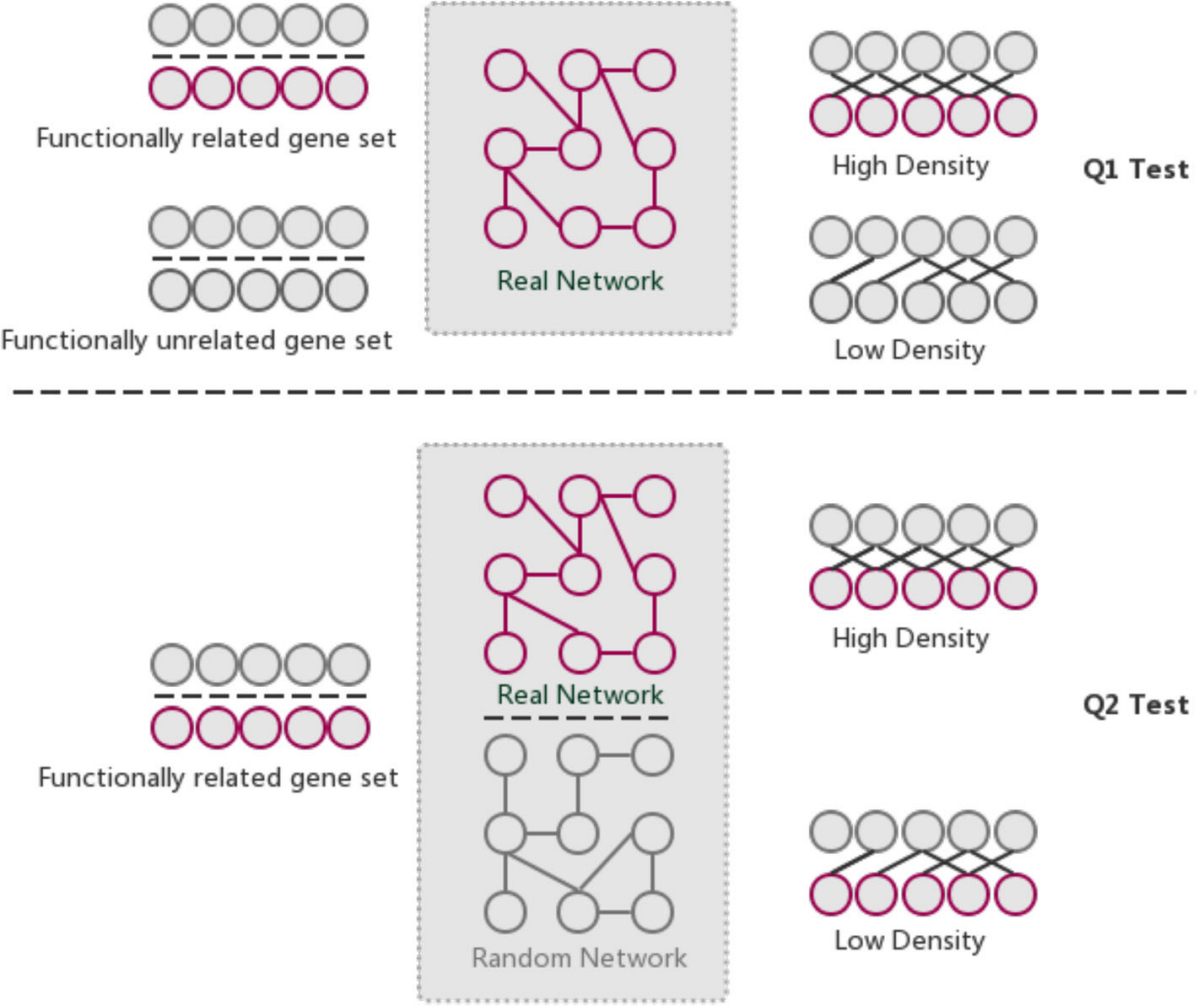
Two hypothesis tests that GSLA used to identify significant functional associations between two gene sets that are biologically meaningful. Q1 tests whether the density of functional associations between two biologically meaningful gene sets is higher than random gene pairs, while Q2 tests whether the strong functional associations observed between two gene sets can only be observed from the biologically correct network, rather than any random interactomes

The GSLA web tool was first developed for the Predicted Arabidopsis Interactome Resource (PAIR) to interpret potential functional impacts of observed SDEG in Arabidopsis [14]. Two testing hypotheses (Q1 and Q2) are used in GSLA to ensure that the reported associations between two *C. elegans* gene sets are significant (Fig. 4). Q1 examines whether the gene association density of inter-gene-sets between two functionally associated gene sets is higher than the background random gene sets. Q2 examines whether the observed high-density gene associations between two gene sets can only be observed in the biologically correct functional gene association network. In other words, the density observed in FIC is higher than the densities observed in random gene association networks consisting of the same genes, with each gene having the same number of neighbours. From a biological perspective, Q1 tests the strength of a functional association between two gene sets. Q2 confirms that the observed strong inter-gene-set functional interaction is the result of a biologically correct functional interactome (i.e., our knowledge of the molecular mechanisms), rather than the result of the gene set compositions. As is well-known, in an interactome, some hub genes have substantially more neighbours than others. Gene sets that include a number of hubs may easily have many inter-gene-set functional interactions with other gene sets. Therefore, Q2 is used to control this confounding factor of gene set composition. Q1 and Q2 are different but complementary tests, both of which can increase the sensitivity and specificity of GSLA and can ensure the biological significance of the functional associations detected between gene sets. The default significance cut-offs for GSLA to report a gene set interaction are density > 0.01 (Q1) and *p* < 0.001 (Q2).

The link for GSLA online service is provided in the web interface of FIC, the functional gene association network of which is used to interpret the functional impacts of observed SDEG in *C. elegans*. The main interface is shown in Fig. 3c. In this instance, we provide five types of *C. elegans* gene IDs for users to query SDEG, including WormBase ID, gene name, UniProt ID, Ensembl gene ID, Ensembl protein ID, and NCBI Entrez ID. We suggest providing SDEG directly in WormBase IDs because WormBase ID is only recognized by the internal server. Therefore, all of the submitted IDs will be automatically mapped to WormBase ID before further computation (Fig. 3d). To avoid loss of information during ID mapping processes, submitting SDEG lists to WormBase ID is better. The criteria of GSLA to report significant functional gene associations (Q1 and Q2 tests, described above) can be optimized by users (Fig. 3c). Before submission, an email address is needed for the results. Utilizing the top 50–200 changed genes is recommended when querying the observed SDEG to obtain optimal functional impact interactions. The analysis parameters are provided at the top ten lines in the result file (Fig. 3e). Below these parameters, a table is presented to show the functionally associated biological processes, functional associations between genes in reported biological processes, and the genes in the query SDEG.

### Using the FIC/GSLA system to re-analyse the *E. coli* HT115 diet dataset

*E. coli* OP50 and *E. coli* HT115 are two standard laboratory diets for *C. elegans* [31]. *E. coli* OP50 is the most commonly used food, while *E. coli* HT115 is typically reared in RNAi-mediated gene knockdown experiments. However, some studies have discovered that *E. coli* OP50, as the most common lab diet for *C. elegans*, will cause a mild, chronic vitamin B12 deficiency [31, 46, 47]. Therefore, Revtovich et al. performed a variety of assays and confirmed a diet of *E. coli* OP50 results in vitamin B12 deficiency, which disrupts mitochondrial homeostasis and decreases the host resistance [31]. Feeding *C. elegans E. coli* HT115 or overexpression of the B12 transporter improved mitochondrial homeostasis and increased resistance. To further explore the molecular mechanisms underlying the diet-induced difference in stress resistance, these researchers performed a genetic analysis to map this phenotype to the methylmalonyl/succinyl-CoA breakdown pathway, where vitamin B12 serves as a cofactor for MMCM-1/MUT. The authors compared the transcriptomic profiles of *C. elegans* with different diets, *E. coli* OP50 and *E. coli* HT115 (GEO database, GSE97678). The results of the microarray analysis showed that the number of differentially regulated genes was relatively small. Only 22 genes were upregulated in *E. coli* OP50 (between 2- and 20-fold). Interestingly, more than half (12 genes) of these 22 upregulated gene-encoded proteins were localized on mitochondria.

**Fig. 5:**
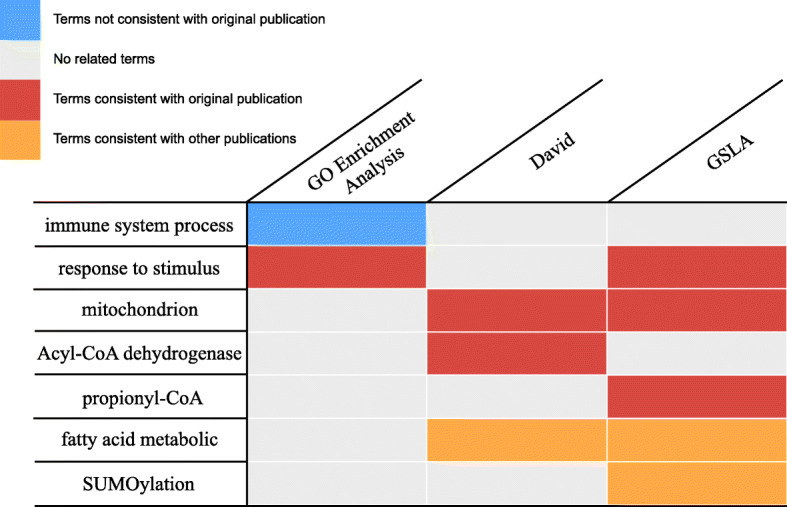
Functional interpretations produced by FIC/GSLA. Compared to GO enrichment analysis and DAVID, the annotations produced by GSLA are more comprehensive and more accurate

In this study, we used the FIC/GSLA system to reanalyse the functional impacts of different diet-induced gene changes. To evaluate whether the prediction tools can obtain biological insights from these changed genes of the microarray data (GEO database, GSE97678) [31], we performed FIC/GSLA, DAVID [12], and GO enrichment analysis [44, 45]. As shown in Fig. 5, GO enrichment analysis reported immune response- and defence response-related pathways (Additional file 6: Table S5). Defence response-related GO terms were consistent with the original publication; however, the authors have clearly documented that diet-mediated sensitivity is independent of innate immunity [31]. The term clustering technology-based tool DAVID reported a total of 43 terms in 7 clusters. Among them, there are 22 GO terms. The top 5 GO terms (ranked by PValue) included the biological processes of mitochondrial and acyl-CoA dehydrogenase-related metabolism that are consistent with those reported in the original article (Additional file 7: Table S6). For comparison, we also used FIC/GSLA to annotate the SDEGs (Additional file 8: Table S7). Eighteen terms covered defence response, mitochondria, and propionyl-CoA pathways (Fig. 5), which are known to be the “functional impact” of the *E. coli* OP50 diet. Moreover, both the DAVID and FIC/GSLA systems reported fatty acid metabolic processes that are consistent with the discoveries by Brooks et al. [48]. In addition, GSLA also found SUMOylation-related biological processes (Fig. 5). In an independent study, Benedetti et al. indeed observed that ubiquitin-like protein 5 (UBL-5) positively regulates chaperone gene expression in response to mitochondrial unfolded proteins [49]. In this case study, the interpretation provided by FIC/GSLA is broader and more accurate, providing new insights for experimental researchers to explore molecular mechanisms, while other widely used enrichment-based tools did not provide similar insights.

## Discussion

Before this work, numerous efforts have been made to build reference interactomes for *C. elegans*. An accurate and comprehensive reference interactome may facilitate the interpretation of gene transcriptional changes to higher-level biological process changes. To date, many *C. elegans* interactome databases have emerged. Some of these interactome databases, e.g., WormBase [16], BioGRID [24], and IntAct [25], collect experimentally reported molecular interactions. Others, such as WormNet [17] and STRING [20], provide predicted molecular interactions. In general, although experimentally reported molecular interactions are considered to be more accurate than predicted molecular interactions, the number of molecular interactions reported by experiments is too low. Based on our estimated size of the *C. elegans* interactome, which is 1.42 × 10^5^, the WormBase interactome database that provides experimental molecular interactions shows the highest coverage with sizes up to 10.35%, which represents 17.15% of the *C. elegans* protein interactome. In fact, due to the false positive experimental molecular interactions, the coverage of the WormBase database will be lower than 10.35%. Such a low coverage may provide limited support for researchers to explain molecular mechanisms during their research. In contrast to the databases with experimental interactions, the databases with predicted molecular interactions may present a high coverage of the true *C. elegans* protein interactomes. STRING is a widely used interactome that contains a large number of predicted interactions. In *C. elegans*, STRING presents 31.89% of predicted interactions, which cover 31.89% of the protein interactome. However, these predicted interactions often have a very high false positive rate, and only 3.46% of them are expected to represent protein interactions. Compared to the currently widely used interactome databases, our newly developed FIC interactome shows better performance with balanced coverage and reliability (21.42% coverage and 29.25% reliability if evaluated as a protein interaction network). In conclusion, FIC is a high-quality reference protein interaction network used to analyse functional gene interactions.

Our high-quality FIC enables GSLA for the interpretation of the observed SDEG in *C. elegans*. FIC has both a high-precision and high-coverage functional interactome, which helps GSLA to report significant functional associations between gene sets. The assessment of GSLA evaluates the density of functional gene associations between individual genes in two gene sets. The interactome of FIC with balanced accuracy will facilitate the successful application of this strategy, while previous interactomes did not satisfy this requirement. We assessed the GSLA interpretation of FIC compared to other interactomes, and FIC showed the best performance (data not shown). The same observation was made when we previously developed high-quality functional interactomes for humans and Arabidopsis.

As mentioned in the introduction, the FIC/GSLA system can interpret the potential functional impacts of the observed SDEG of *C. elegans*. Therefore, the FIC/GSLA system extends the availability of current enrichment-based tools to summarize SDEG into known biological processes. In some cases, even when no established biological concept can accurately explain the observed SDEG, the FIC/GSLA system may still be able to interpret the observed gene transcriptional changes and to connect the related physiologies. Moreover, the functional association resource provided in FIC is a useful reference for researchers to elucidate the molecular mechanisms of their genes of interest.

## Conclusions

Thus, predicted FIC is a reliable and high-quality resource for querying functional associations between genes of *C. elegans*, as it can help researchers understand the molecular mechanisms of certain genes. Based on the functional gene association network of FIC, GSLA was developed to facilitate the interpretation of potential functional impacts from the observed differentially expressed genes, especially when no established biological concept is available to describe the observed SDEG. A case study of the FIC/GSLA system shows that the reported annotations are more comprehensive and concise annotations compared to the other widely used annotation tools, including PANTHER and DAVID.

## Supplementary information

**Additional file 1: Table S1.** Number of protein-protein interactions and participating unique proteins from three databases.

**Additional file 2: Table S2.** Functional association evidence and computing methods.

**Additional file 3: Table S3.** Assessment of feature quality.

**Additional file 4: Figure S1.** Receiver operating characteristic (ROC) curves of 36 feature values. Features with areas under the curve (AUC) above 0.6 were selected for use in the SVM model to predict functional interactions between genes.

**Additional file 5: Table S4.** Evaluation of the predicted interactions in different datasets.

**Additional file 6: Table S5.** Annotations produced by the GO enrichment analysis tool for the transcriptionally changed genes.

**Additional file 7: Table S6.** Annotations produced by DAVID for the transcriptionally changed genes.

**Additional file 8: Table S7.** Annotations produced by FIC/GSLA for the transcriptionally changed genes.

## Data Availability

The predicted interactome FIC and its associated GSLA web tool are available at http://worm.biomedtzc.cn.
