# Supplementary material for: Predicted functional interactome of Caenorhabditis elegans and a web tool for the functional interpretation of differentially expressed genes
Source: Biol Direct. 2020 Oct 19;15:20. doi: 10.1186/s13062-020-00271-6 (PMC7574172; doi:10.1186/s13062-020-00271-6)
Supplement: Supplementary file 1 — Additional file 1: Table S1. Number of protein-protein interactions and participating unique proteins from three databases. [file 13062_2020_271_MOESM1_ESM.pdf]

Supplementary Table s1. Number of protein-protein interactions and participating unique proteins from three databases.

Database	All interactions		High confidence interactions*		Date
	Number of interactions	Number of Proteins	Number of interactions	Number of Proteins	
BioGrid	8608	3885	442	497	Dec 25th, 2017
Intact	15586	5031	757	828	Dec 12th, 2017
WormBase	14698	2709	5306	2582	Nov 13th,2017
Total	18890	6195	5606	2896	

*High confidence interactions: interactions reported in more than two independent studies, at least one of which is low-throughput experiment.
