# Supplementary material for: Predicted functional interactome of Caenorhabditis elegans and a web tool for the functional interpretation of differentially expressed genes
Source: Biol Direct. 2020 Oct 19;15:20. doi: 10.1186/s13062-020-00271-6 (PMC7574172; doi:10.1186/s13062-020-00271-6)
Supplement: Supplementary file 2 — Additional file 2: Table S2. Functional association evidence and computing methods. [file 13062_2020_271_MOESM2_ESM.pdf]

Supplementary Table s2. Functional association evidence and computing methods.

Feature	Data source/ Data set		Statistics
Shared Annotation	GOC	Cellular component<br>Biological process<br>Molecular function	Maximum Shared Annotation Score
Homologous Interactions	Inparanoid Score		Maximum
Domain Interaction	IDDI	3DID<br>IPFAM<br>PINS<br>TW<br>HIMAP<br>DOMAINGA<br>PVALUE<br>IPPRI<br>RCDP<br>DIPD<br>RDFF<br>DPEA<br>ME<br>PE<br>DIMA_STRING<br>KGIDDI<br>LLZ<br>GPE<br>DIMA_DPROF<br>APMM<br>TOPDOWN<br>DIMA_DPEA<br>INTERDOM	Maximum Shared Domain Score
Co-localization	Compartments	Knowledge channel<br>Experiments channel<br>Text mining channel<br>Predictions channel	Cosine Similarity Score
Co-expression	COXPRESdb	Microarray<br>RNASeq	Pearson's Correlation Score
Phylogenetic Profile	DIOPT		Mutual Information Score<br>Pearson's Correlation Score<br>Tanimato Correlation Score
