# Supplementary material for: Predicted functional interactome of Caenorhabditis elegans and a web tool for the functional interpretation of differentially expressed genes
Source: Biol Direct. 2020 Oct 19;15:20. doi: 10.1186/s13062-020-00271-6 (PMC7574172; doi:10.1186/s13062-020-00271-6)
Supplement: Supplementary file 3 — Additional file 3: Table S3. Assessment of feature quality. [file 13062_2020_271_MOESM3_ESM.pdf]

Supplementary Table s3.Assessment of feature quality.

Evidence	type Feature	AUC of	AUC > 0.6
Shared Annotation	biological_process_value	0.828	Yes
	molecular_function_value	0.637	Yes
	cellular_component_value	0.751	Yes
Coexpression	RNAseq_value	0.814	Yes
	Microarray_value	0.717	Yes
Phylogenetic profile	phyprofile_tanimato_valu	0.755	Yes
	phyprofile_pearson_value	0.749	Yes
	phyprofile_mutual_value	0.626	Yes
Colocation	integrated_value	0.615	Yes
	knowledge_value	0.554	No
	predictions_value	0.589	No
	textmining_value	0.517	No
Domain Interactions	INTERDOM_value	0.663	Yes
	PINS_value	0.538	No
	LLZ_value	0.514	No
	RDFF_value	0.535	No
	DIPD_value	0.527	No
	3DID_value	0.551	No
	IPPRI_value	0.513	No
	ME_value	0.548	No
	KGIDDI_value	0.547	No
	APMM_value	0.585	No
	IPFAM_value	0.542	No
	DOMAINGA_value	0.503	No
	RCDP_value	0.514	No
	TOPDOWN_value	0.565	No
	DIMA_DPEA_value	0.557	No
	GPE_value	0.581	No
	DIMA_DPROF_value	0.514	No
	PVALUE_value	0.510	No
	TW_value	0.502	No
	HIMAP_value	0.505	No
	PE_value	0.531	No
	DIMA_STRING_value	0.531	No
	DPEA_value	0.510	No
Homologous interactions	inparanoid_value	0.523	No
