# Supplementary figures and images for: Predicted functional interactome of Caenorhabditis elegans and a web tool for the functional interpretation of differentially expressed genes
Source: Biol Direct. 2020 Oct 19;15:20. doi: 10.1186/s13062-020-00271-6 (PMC7574172; doi:10.1186/s13062-020-00271-6)

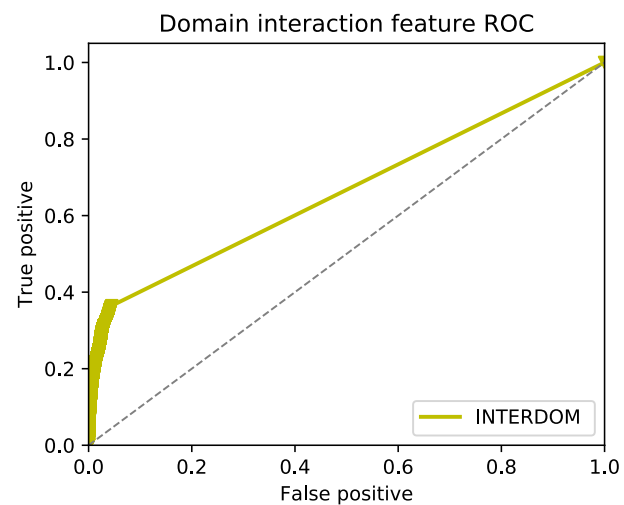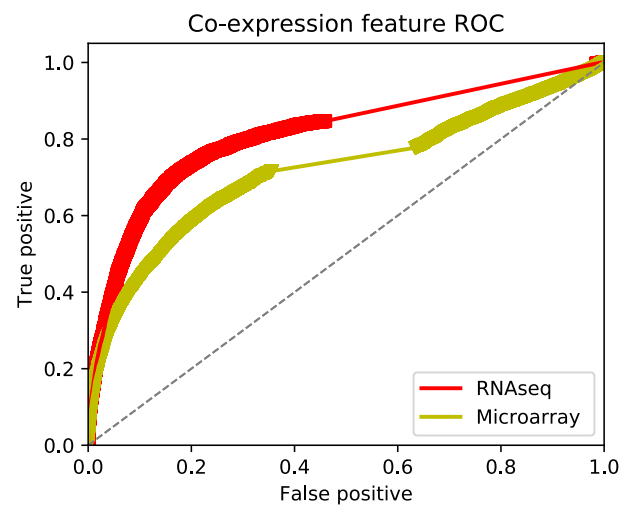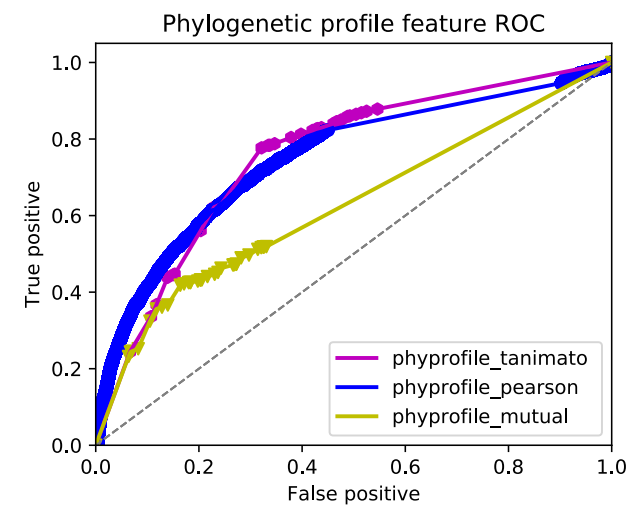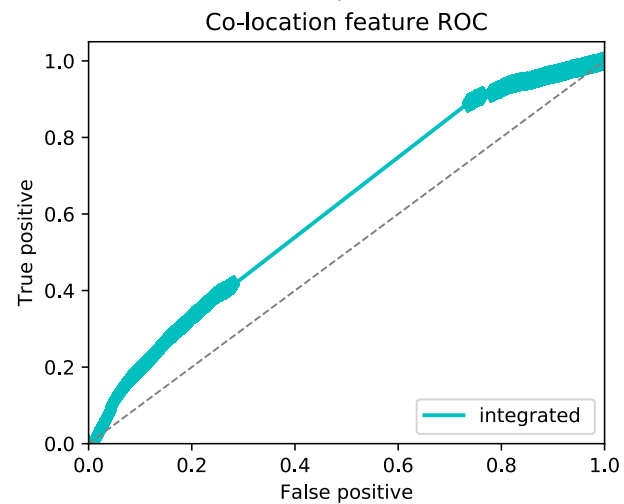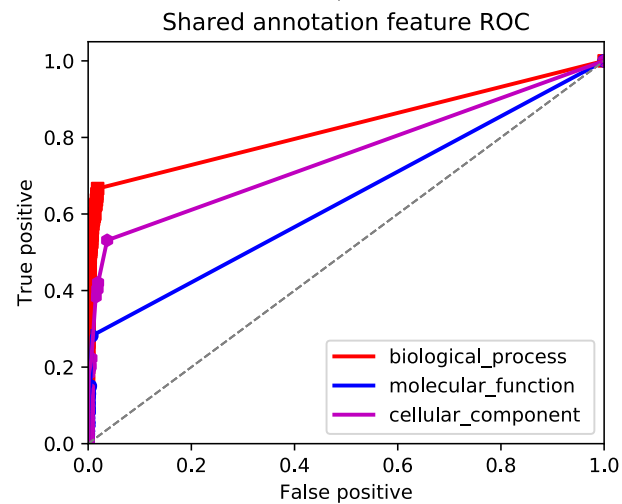

Supplement: Supplementary file 4 — Additional file 4: Figure S1. Receiver operating characteristic (ROC) curves of 36 feature values. Features with areas under the curve (AUC) above 0.6 were selected for use in the SVM model to predict functional interactions between genes. [file 13062_2020_271_MOESM4_ESM.pdf]
