# Supplementary material for: Predicted functional interactome of Caenorhabditis elegans and a web tool for the functional interpretation of differentially expressed genes
Source: Biol Direct. 2020 Oct 19;15:20. doi: 10.1186/s13062-020-00271-6 (PMC7574172; doi:10.1186/s13062-020-00271-6)
Supplement: Supplementary file 5 — Additional file 5: Table S4. Evaluation of the predicted interactions in different datasets. [file 13062_2020_271_MOESM5_ESM.pdf]

Supplementary Table s4.Evaluation of the predicted interactions in different datasets.

Network	Sensitivity	Reliability
**FIC**	**21.42%**	**29.25%**
MIST	19.46%	4.40%
STRING	31.89%	1.37%
WormNet	31.28%	5.83%
