# Supplementary material for: Predicted functional interactome of Caenorhabditis elegans and a web tool for the functional interpretation of differentially expressed genes
Source: Biol Direct. 2020 Oct 19;15:20. doi: 10.1186/s13062-020-00271-6 (PMC7574172; doi:10.1186/s13062-020-00271-6)
Supplement: Supplementary file 6 — Additional file 6: Table S5. Annotations produced by the GO enrichment analysis tool for the transcriptionally changed genes. [file 13062_2020_271_MOESM6_ESM.pdf]

Supplementary Table s5. Annotations produced by the GO enrichment analysis tool for the transcriptionally changed genes.

Analysis Type:	PANTHER Overrepresentation Test (Released 20190711)						
Annotation Version and Release Date:	GO Ontology database Released 2019-07-03						
Analyzed List:	upload_1 (Caenorhabditis elegans)						
Reference List:	Caenorhabditis elegans (all genes in database)						
Test Type:	FISHER						
Correction:	FDR						
GO biological process complete	Caenorhabditis elegans - REFLIST (19921)	upload_1 (127)	upload_1 (expected)	upload_1 (over/under)	upload_1 (fold Enrichment)	upload_1 (raw P-value)	upload_1 (FDR)
innate immune response (GO:0045087)	223	10	1.42	+	7.03	2.37E-06	1.48E-02
immune response (GO:0006955)	225	10	1.43	+	6.97	2.55E-06	7.97E-03
immune system process (GO:0002376)	230	10	1.47	+	6.82	3.08E-06	6.42E-03
defense response to other organism (GO:0098542)	291	11	1.86	+	5.93	3.57E-06	5.57E-03
response to external biotic stimulus (GO:0043207)	293	11	1.87	+	5.89	3.80E-06	4.75E-03
response to other organism (GO:0051707)	293	11	1.87	+	5.89	3.80E-06	3.96E-03
response to biotic stimulus (GO:0009607)	293	11	1.87	+	5.89	3.80E-06	3.39E-03
defense response (GO:0006952)	296	11	1.89	+	5.83	4.18E-06	3.26E-03
multi-organism process (GO:0051704)	589	14	3.75	+	3.73	2.91E-05	2.02E-02
