# Supplementary material for: Predicted functional interactome of Caenorhabditis elegans and a web tool for the functional interpretation of differentially expressed genes
Source: Biol Direct. 2020 Oct 19;15:20. doi: 10.1186/s13062-020-00271-6 (PMC7574172; doi:10.1186/s13062-020-00271-6)
Supplement: Supplementary file 7 — Additional file 7: Table S6. Annotations produced by DAVID for the transcriptionally changed genes. [file 13062_2020_271_MOESM7_ESM.pdf]

Supplementary Table s6. Annotations produced by DAVID for the transcriptionally changed genes.

Annotation Cluster 1 Enrichment Score: 3.328352017165468

Category	Term	Count	%	PValue	Genes	List Total	Pop Hits	Pop Total	Fold Enrichment	Bonferroni	Benjamini	FDR	Included for comparison (Top 5 significance)
GOTERM_BP_DIRECT	GO:0055114~oxidation-reduction process	9	19.56521739	8.03E-05	Y38F1A.6, CYP-33C7, DHS-20, DHS-25, F54D5.12, ACOX-2, PDHB-1, ACDH-2, ACDH-1	34	522	11627	5.896044625	0.005764776	0.005764776	0.083545402	Y
GOTERM_MF_DIRECT	GO:0016491~oxidoreductase activity	8	17.39130435	2.75E-04	Y38F1A.6, CYP-33C7, DHS-20, DHS-25, F54D5.12, PDHB-1, ACDH-2, ACDH-1	31	422	9479	5.796667176	0.016115775	0.016115775	0.274605561	Y
UP_KEYWORDS	Oxidoreductase	5	10.86956522	0.004681596	Y38F1A.6, CYP-33C7, PDHB-1, ACDH-2, ACDH-1	46	306	20248	7.1923842	0.212838674	0.076675025	4.438096986	N

Annotation Cluster 2 Enrichment Score: 2.7288590586119073

Category	Term	Count	%	PValue	Genes	List Total	Pop Hits	Pop Total	Fold Enrichment	Bonferroni	Benjamini	FDR	Included for comparison (Top 5 significance)
UP_KEYWORDS	Transit peptide	5	10.86956522	1.75E-04	F09F7.4, Y38F1A.6, KAT-1, C05C10.3, PDHB-1	46	126	20248	17.46721877	0.008877313	0.008877313	0.168998754	N
UP_KEYWORDS	Mitochondrion	6	13.04347826	1.94E-04	F09F7.4, Y38F1A.6, KAT-1, C05C10.3, PDHB-1, DDP-1	46	242	20248	10.9134028	0.009823893	0.00492407	0.187091226	N
GOTERM_CC_DIRECT	GO:0005739~mitochondrion	7	15.2173913	3.16E-04	F09F7.4, Y38F1A.6, KAT-1, C05C10.3, PDHB-1, DDP-1, ACDH-1	27	424	11373	6.954140461	0.008184811	0.008184811	0.258887459	Y
KEGG_PATHWAY	cel00280:Valine, leucine and isoleucine degradation	3	6.52173913	0.005800102	F09F7.4, KAT-1, C05C10.3	10	40	3022	22.665	0.135344994	0.070131726	4.609816922	N
UP_SEQ_FEATURE	transit peptide:Mitochondrion	3	6.52173913	0.020357392	Y38F1A.6, C05C10.3, PDHB-1	9	97	3383	11.62542955	0.376902062	0.376902062	15.04301943	N
KEGG_PATHWAY	cel01200:Carbon metabolism	3	6.52173913	0.03353638	F09F7.4, KAT-1, PDHB-1	10	100	3022	9.066	0.573776133	0.247431698	24.17599014	N

Annotation Cluster 3 Enrichment Score: 2.4925348191656416

Category	Term	Count	%	PValue	Genes	List Total	Pop Hits	Pop Total	Fold Enrichment	Bonferroni	Benjamini	FDR	Included for comparison (Top 5 significance)
GOTERM_MF_DIRECT	GO:0050660~flavin adenine dinucleotide binding	4	8.695652174	9.54E-04	F54D5.12, ACOX-2, ACDH-2, ACDH-1	31	62	9479	19.72736733	0.054754382	0.027762571	0.948534519	Y
INTERPRO	IPR006091:Acyl-CoA oxidase/dehydrogenase, central domain	3	6.52173913	0.001437959	ACOX-2, ACDH-2, ACDH-1	40	21	14476	51.7	0.122737	0.122737	1.55653807	N
INTERPRO	IPR013786:Acyl-CoA dehydrogenase/oxidase, N-terminal	3	6.52173913	0.001579068	ACOX-2, ACDH-2, ACDH-1	40	22	14476	49.35	0.133946616	0.069380108	1.708091019	N
INTERPRO	IPR009100:Acyl-CoA dehydrogenase/oxidase	3	6.52173913	0.001726517	ACOX-2, ACDH-2, ACDH-1	40	23	14476	47.20434783	0.145508616	0.051066244	1.86622791	N
INTERPRO	IPR009075:Acyl-CoA dehydrogenase/oxidase C-terminal	3	6.52173913	0.001726517	ACOX-2, ACDH-2, ACDH-1	40	23	14476	47.20434783	0.145508616	0.051066244	1.86622791	N
GOTERM_BP_DIRECT	GO:0033539~fatty acid beta-oxidation using acyl-CoA dehydrogenase	3	6.52173913	0.002073493	ACOX-2, ACDH-2, ACDH-1	34	24	11627	42.74632353	0.13881548	0.048595054	2.137319586	Y
GOTERM_MF_DIRECT	GO:0003995~acyl-CoA dehydrogenase activity	3	6.52173913	0.002350766	ACOX-2, ACDH-2, ACDH-1	31	23	9479	39.88359046	0.129648786	0.045231285	2.322794637	N
GOTERM_MF_DIRECT	GO:0052890~oxidoreductase activity, acting on the CH-CH group of donors, with a flavin as acceptor	3	6.52173913	0.002559438	ACOX-2, ACDH-2, ACDH-1	31	24	9479	38.22177419	0.140324639	0.037094605	2.526619376	N
GOTERM_BP_DIRECT	GO:0055088~lipid homeostasis	3	6.52173913	0.002819719	ACOX-2, ACDH-2, ACDH-1	34	28	11627	36.63970588	0.183971943	0.049556571	2.896353094	N
GOTERM_MF_DIRECT	GO:0000062~fatty-acyl-CoA binding	3	6.52173913	0.005399791	ACOX-2, ACDH-2, ACDH-1	31	35	9479	26.20921659	0.273452138	0.061891986	5.263181125	N
GOTERM_MF_DIRECT	GO:0016627~oxidoreductase activity, acting on the CH-CH group of donors	3	6.52173913	0.005399791	ACOX-2, ACDH-2, ACDH-1	31	35	9479	26.20921659	0.273452138	0.061891986	5.263181125	N

UP_KEYWORDS	FAD	3	6.52173913	0.01088915	ACOX-2, ACDH-2, ACDH-1	46	71	20248	18.59889773	0.427871919	0.130292298	10.05024929	N
UP_KEYWORDS	Flavoprotein	3	6.52173913	0.012714155	ACOX-2, ACDH-2, ACDH-1	46	77	20248	17.14963298	0.479298979	0.122357419	11.6430114	N
GOTERM_MF_DIRECT	GO:0009055~electron carrier activity	3	6.52173913	0.013873808	ACOX-2, ACDH-2, ACDH-1	31	57	9479	16.09337861	0.561451986	0.097905275	13.0216919	N
Annotation Cluster 4	Enrichment Score: 0.6838008427897013												
Category	Term	Count	%	PValue	Genes	List Total	Pop Hits	Pop Total	Fold Enrichment	Bonferroni	Benjamini	FDR	Included for comparison (Top 5 significance)
UP_KEYWORDS	Metal-binding	7	15.2173913	0.057121731	CYP-33C7, NHR-49, KAT-1, NHR-202, C05C8.7, SAMS-1, DDP-1	46	1251	20248	2.46300351	0.950199127	0.393441313	43.39111389	N
GOTERM_MF_DIRECT	GO:0046872~metal ion binding	8	17.39130435	0.182187516	Y38F1A.6, CYP-33C7, NHR-49, KAT-1, NHR-202, C05C8.7, SAMS-1, DDP-1	31	1492	9479	1.639539912	0.999992976	0.732455078	86.57928636	N
UP_KEYWORDS	Zinc	4	8.695652174	0.241142778	NHR-49, NHR-202, C05C8.7, DDP-1	46	765	20248	2.301562944	0.999999227	0.866069181	93.07096667	N
GOTERM_MF_DIRECT	GO:0008270~zinc ion binding	3	6.52173913	0.733166139	NHR-49, NHR-202, C05C8.7	31	800	9479	1.146653226	1	0.999588072	99.99981356	N
Annotation Cluster 5	Enrichment Score: 0.2951462782448386												
Category	Term	Count	%	PValue	Genes	List Total	Pop Hits	Pop Total	Fold Enrichment	Bonferroni	Benjamini	FDR	Included for comparison (Top 5 significance)
UP_KEYWORDS	Transcription regulation	3	6.52173913	0.324534552	NHR-49, NHR-202, MDT-18	46	523	20248	2.524898163	0.999999998	0.891752523	97.75308516	N
UP_KEYWORDS	Transcription	3	6.52173913	0.350667096	NHR-49, NHR-202, MDT-18	46	555	20248	2.379318449	1	0.889443624	98.46603247	N
GOTERM_BP_DIRECT	GO:0006351~transcription, DNA-templated	3	6.52173913	0.491654767	NHR-49, NHR-202, MDT-18	34	576	11627	1.781096814	1	0.995540666	99.91260729	N
GOTERM_BP_DIRECT	GO:0006355~regulation of transcription, DNA-templated	3	6.52173913	0.643518188	NHR-49, NHR-202, MDT-18	34	758	11627	1.3534456	1	0.998830821	99.99782599	N
GOTERM_CC_DIRECT	GO:0005634~nucleus	5	10.86956522	0.647519209	F22B8.7, NHR-49, CIR-1, NHR-202, MDT-18	27	1888	11373	1.115524953	1	0.999881102	99.98067328	N
UP_KEYWORDS	Nucleus	3	6.52173913	0.726930071	NHR-49, NHR-202, MDT-18	46	1136	20248	1.162431108	1	0.993855889	99.99964801	N
Annotation Cluster 6	Enrichment Score: 0.06999528231750873												
Category	Term	Count	%	PValue	Genes	List Total	Pop Hits	Pop Total	Fold Enrichment	Bonferroni	Benjamini	FDR	Included for comparison (Top 5 significance)
UP_KEYWORDS	Transport	3	6.52173913	0.482496042	FOLT-2, HRG-1, DDP-1	46	725	20248	1.821409295	1	0.95283718	99.82923034	N
GOTERM_BP_DIRECT	GO:0006810~transport	3	6.52173913	0.680112474	FOLT-2, HRG-1, DDP-1	34	810	11627	1.266557734	1	0.998928521	99.9992959	N
UP_KEYWORDS	Membrane	8	17.39130435	0.993158238	DCAR-1, FOLT-2, STR-7, F22E5.1, CNC-7, HRG-1, DDP-1, COL-81	46	6152	20248	0.572397806	1	0.999999956	100	N
UP_KEYWORDS	Transmembrane helix	7	15.2173913	0.996853018	DCAR-1, FOLT-2, STR-7, F22E5.1, CNC-7, HRG-1, COL-81	46	5972	20248	0.51594397	1	0.999999989	100	N
UP_KEYWORDS	Transmembrane	7	15.2173913	0.996872301	DCAR-1, FOLT-2, STR-7, F22E5.1, CNC-7, HRG-1, COL-81	46	5975	20248	0.515684919	1	0.999999969	100	N
GOTERM_CC_DIRECT	GO:0016021~integral component of membrane	7	15.2173913	0.999569483	DCAR-1, FOLT-2, STR-7, F22E5.1, CNC-7, HRG-1, COL-81	27	6032	11373	0.488818892	1	1	100	N
GOTERM_CC_DIRECT	GO:0016020~membrane	7	15.2173913	0.999669479	DCAR-1, FOLT-2, F22E5.1, CNC-7, HRG-1, DDP-1, COL-81	27	6114	11373	0.48226293	1	1	100	N
Annotation Cluster 7	Enrichment Score: 0.0671966339605672												
Category	Term	Count	%	PValue	Genes	List Total	Pop Hits	Pop Total	Fold Enrichment	Bonferroni	Benjamini	FDR	Included for comparison (Top 5 significance)

GOTERM_BP_DIRECT	GO:0000003~reproduction	6	13.04347826	0.777221624	NHR-49, CIR-1, C05C8.7, SAMS-1, MDT-18, F55F8.2	34	2205	11627	0.930532213	1		0.999755533	99.9999837	N
GOTERM_BP_DIRECT	GO:0002119~nematode larval development	5	10.86956522	0.841411407	NHR-49, CIR-1, C05C8.7, MDT-18, F55F8.2	34	1993	11627	0.857929223	1		0.99992289	99.99999953	N
GOTERM_BP_DIRECT	GO:0009792~embryo development ending in birth or egg hatching	6	13.04347826	0.961295319	F09F7.4, NHR-49, CIR-1, C05C8.7, PDHB-1, MDT-18	34	3091	11627	0.663805736	1		0.999999558	100	N
