# Supplementary material for: Predicted functional interactome of Caenorhabditis elegans and a web tool for the functional interpretation of differentially expressed genes
Source: Biol Direct. 2020 Oct 19;15:20. doi: 10.1186/s13062-020-00271-6 (PMC7574172; doi:10.1186/s13062-020-00271-6)
Supplement: Supplementary file 8 — Additional file 8: Table S7. Annotations produced by FIC/GSLA for the transcriptionally changed genes. [file 13062_2020_271_MOESM8_ESM.pdf]

Supplementary Table s7. Annotations produced by FIC/GSLA for the transcriptionally changed genes.

Job Number: GSLA07294387667044
Cutoff used:
Q1: density >= 0.01
Q2: p <= 0
Meaning:
Q1: inter-geneset interaction density is greater than expected.
Q2: the observed interaction density can only be observed in the biologically correct interactome topology.
Categories selected: GC Panther pathway   Reactome pathway
Species: Caenorhabditis elegans
Interaction dataset selected: FIC

#Subjob Number: GSLA07294387667044_0
#Description: #diet-TOP50
#Quaried GeneSet: clec-55,clec-52,nhr-202,asp-10,F54D5.12,nhr-49,str-7,dhs-20,F15E6.4,pdhb-1,cth-1,C54C8.12,prx-11,acdh-1,acdh-2,C05C10.3,C09B8.4,F22B8.7,F22E5.1,folt-2,H39E23.3,hrg-1,ilys-2,kat-1,sams-1,T07D3.9,Y37D8A.4,dhs-25,F20G2.1,farl-11,acox-2,C05C8.7,cir-1,cnc-7,cyp-33C7,dcar-1,F35E12.5,F44G3.2,tag-38,C32H11.4,ddp-1,col-81,Y38F1A.6,M03A1.8///dao-2,mdt-18,F09F7.4,F55F8.2,acdh-2,C24B9.3

Term type	Term	Description	Term size	P value	Density	Interaction number	Overlap gene number	Overlap gene(s)	Interactions
Reactome pathway	R-CEL-389887	Beta-oxidation of pristanoyl-CoA	11	0	0.01446	7	0		nhr-49-WBGene00019060|nhr-49-WBGene00008167|nhr-49-WBGene00010336|kat-1-WBGene00008167|kat-1-WBGene00008565|kat-1-WBGene00008564|T07D3.9-WBGene00016511
Panther pathway	P02737	Cysteine biosynthesis	5	0	0.02272	5	0		cth-1-WBGene00013866|cth-1-WBGene00019962|cth-1-WBGene00019096|cth-1-WBGene00010759|cth-1-WBGene00007653
GO biological process	GO:0050832	defense response to fungus	24	0	0.02556	27	1	dcar-1	nhr-49-WBGene00006575|nhr-49-WBGene00010700|nhr-49-WBGene00006599|nhr-49-WBGene00004055|nhr-49-WBGene00001674|dcar-1-WBGene00010556|dcar-1-WBGene00000253|dcar-1-WBGene00006575|dcar-1-WBGene00000555|dcar-1-WBGene00000556|dcar-1-WBGene00003771|dcar-1-WBGene00015303|dcar-1-WBGene00000558|dcar-1-WBGene00010700|dcar-1-WBGene00044900|dcar-1-WBGene00006599|dcar-1-WBGene00004860|dcar-1-WBGene00000900|dcar-1-WBGene00003096|dcar-1-WBGene00004857|dcar-1-WBGene00004055|dcar-1-WBGene00000018|dcar-1-WBGene00004034|dcar-1-WBGene00000936|dcar-1-WBGene00003769|dcar-1-WBGene00000560|dcar-1-WBGene00001674
GO biological process	GO:0019217	regulation of fatty acid metabolic process	11	0	0.02685	13	1	nhr-49	nhr-202-WBGene00003670|nhr-202-nhr-49|nhr-49-WBGene00006518|nhr-49-WBGene00015497|nhr-49-WBGene00016610|nhr-49-WBGene00014054|nhr-49-WBGene00021313|nhr-49-WBGene00003670|nhr-49-WBGene00002210|nhr-49-nhr-49|nhr-49-WBGene00007016|dcar-1-WBGene00016610|dcar-1-WBGene00021313

Reactome pathway	R-CEL-77286	mitochondrial fatty acid beta-oxidation of saturated fatty acids	7	0	0.01948	6	0		nhr-49-WBGene00020366|nhr-49-WBGene00020347|kat-1-WBGene00001155|kat-1-WBGene00015125|kat-1-WBGene00020366|kat-1-WBGene00020347
GO biological process	GO:0009620	response to fungus	25	0	0.02454	27	1	dcar-1	nhr-49-WBGene00006575|nhr-49-WBGene00006599|nhr-49-WBGene00001674|nhr-49-WBGene00010700|nhr-49-WBGene00004055|dcar-1-WBGene00000253|dcar-1-WBGene00006575|dcar-1-WBGene00000555|dcar-1-WBGene00000556|dcar-1-WBGene00000558|dcar-1-WBGene00006599|dcar-1-WBGene00004860|dcar-1-WBGene00000900|dcar-1-WBGene00004857|dcar-1-WBGene00000018|dcar-1-WBGene00000560|dcar-1-WBGene00001674|dcar-1-WBGene00010556|dcar-1-WBGene00003771|dcar-1-WBGene00015303|dcar-1-WBGene00010700|dcar-1-WBGene00044900|dcar-1-WBGene00003096|dcar-1-WBGene00004055|dcar-1-WBGene00004034|dcar-1-WBGene00000936|dcar-1-WBGene00003769
GO biological process	GO:0045923	positive regulation of fatty acid metabolic process	8	0	0.02556	9	0		nhr-202-WBGene00003670|nhr-49-WBGene00006518|nhr-49-WBGene00015497|nhr-49-WBGene00016610|nhr-49-WBGene00014054|nhr-49-WBGene00021313|nhr-49-WBGene00003670|dcar-1-WBGene00016610|dcar-1-WBGene00021313
GO biological process	GO:0044272	sulfur compound biosynthetic process	11	0	0.01859	9	0		cth-1-WBGene00013866|cth-1-WBGene00019962|cth-1-WBGene00010759|cth-1-WBGene00007653|C05C8.7-WBGene00005024|farl-11-WBGene00005021|farl-11-WBGene00005024|farl-11-WBGene00005025|farl-11-WBGene00005026
GO biological process	GO:0006024	glycosaminoglycan biosynthetic process	7	0	0.02272	7	0		C05C8.7-WBGene00005024|farl-11-WBGene00005020|farl-11-WBGene00005021|farl-11-WBGene00005022|farl-11-WBGene00005024|farl-11-WBGene00005025|farl-11-WBGene00005026
Reactome pathway	R-CEL-4090294	SUMOylation of intracellular receptors	17	0	0.02272	17	0		nhr-202-WBGene00003607|nhr-202-WBGene00003602|nhr-49-WBGene00004786|nhr-49-WBGene00000908|nhr-49-WBGene00003675|nhr-49-WBGene00003623|nhr-49-WBGene00022374|nhr-49-WBGene00003602|acdh-1-WBGene00003622|dcar-1-WBGene00004786|dcar-1-WBGene00003675|dcar-1-WBGene00006790|dcar-1-WBGene00016126|dcar-1-WBGene00003629|dcar-1-WBGene00003623|dcar-1-WBGene00022374|dcar-1-WBGene00003602

GO biological process	GO:0006631	fatty acid metabolic process	17	0	0.0147	11	0		sams-1-WBGene00000991|sams-1-WBGene00013284|nhr-49-WBGene00009221|nhr-49-WBGene00001397|nhr-49-WBGene00001398|nhr-49-WBGene00001399|nhr-49-WBGene00016610|nhr-49-WBGene00001395|cyp-33C7-WBGene00018334|kat-1-WBGene00001153|dcar-1-WBGene00016610
GO biological process	GO:0005765	lysosomal membrane	10	0	0.0159	7	1	hrg-1	hrg-1-WBGene00001371|hrg-1-WBGene00007979|hrg-1-WBGene00001819|hrg-1-WBGene00003053|hrg-1-WBGene00001814|hrg-1-WBGene00021546|hrg-1-WBGene00008052
Reactome pathway	R-CEL-383280	Nuclear Receptor transcription pathway	57	0	0.02392	60	1	nhr-49	nhr-202-WBGene00016975|nhr-202-WBGene00007547|nhr-202-WBGene00019816|nhr-202-WBGene00015705|nhr-202-WBGene00003630|nhr-202-WBGene00003637|nhr-202-nhr-49|nhr-202-WBGene00008221|nhr-202-WBGene00011097|nhr-202-WBGene00011100|nhr-202-WBGene00003691|nhr-202-WBGene00003605|nhr-202-WBGene00003690|nhr-202-WBGene00003607|nhr-202-WBGene00003609|nhr-202-WBGene00003600|nhr-202-WBGene00003602|nhr-202-WBGene00003706|nhr-202-WBGene00003704|nhr-202-WBGene00003659|nhr-202-WBGene00003658|nhr-202-WBGene00003710|nhr-202-WBGene00003654|nhr-202-WBGene00003616|nhr-202-WBGene00003613|nhr-202-WBGene00003719|nhr-202-WBGene00022423|nhr-202-WBGene00003627|nhr-202-WBGene00003628|nhr-202-WBGene00003726|nhr-202-WBGene00003727|nhr-49-WBGene00015497|nhr-49-WBGene00003675|nhr-49-WBGene00007547|nhr-49-WBGene00003630|nhr-49-WBGene00003637|nhr-49-nhr-49|nhr-49-WBGene00003650|nhr-49-WBGene00003690|nhr-49-WBGene00003609|nhr-49-WBGene00022374|nhr-49-WBGene00003600|nhr-49-WBGene00003602|nhr-49-WBGene00000908|nhr-49-WBGene00003708|nhr-49-WBGene00003654|nhr-49-WBGene00003616|nhr-49-

GO biological process	GO:0002009	morphogenesis of an epithelium	50	0	0.01772	39	1	farl-11	C05C8.7-WBGene00005024|farl-11-WBGene00000395|farl-11-WBGene00000390|farl-11-WBGene00000961|farl-11-WBGene00001330|farl-11-WBGene00001165|farl-11-WBGene00006876|farl-11-WBGene00003258|farl-11-WBGene00014153|farl-11-WBGene00005020|farl-11-WBGene00005021|farl-11-WBGene00005022|farl-11-WBGene00000168|farl-11-WBGene00005023|farl-11-WBGene00005024|farl-11-WBGene00005025|farl-11-WBGene00005026|farl-11-WBGene00006868|farl-11-WBGene00006869|farl-11-WBGene00001687|farl-11-WBGene00016354|farl-11-WBGene00006437|farl-11-WBGene00019030|farl-11-WBGene00010631|farl-11-WBGene00000199|farl-11-WBGene00002181|farl-11-WBGene00000200|farl-11-WBGene00006957|farl-11-WBGene00000204|farl-11-WBGene00000205|farl-11-WBGene00000203|farl-11-WBGene00013355|farl-11-WBGene00006961|farl-11-WBGene00021170|farl-11-WBGene00003241|farl-11-WBGene00004749|farl-11-WBGene00007561|farl-11-WBGene00001333|farl-11-WBGene00001526
GO biological process	GO:1901244	positive regulation of transcription from RNA polymerase II promoter involved in defense response to fungus	9	0	0.04292	17	0		nhr-49-WBGene00004055|nhr-49-WBGene00004758|nhr-49-WBGene00010700|nhr-49-WBGene00010251|nhr-49-WBGene00077712|nhr-49-WBGene00006599|nhr-49-WBGene00003822|nhr-49-WBGene00001674|dcar-1-WBGene00004055|dcar-1-WBGene00004758|dcar-1-WBGene00010700|dcar-1-WBGene00010251|dcar-1-WBGene00001251|dcar-1-WBGene00077712|dcar-1-WBGene00006599|dcar-1-WBGene00003822|dcar-1-WBGene00001674
GO biological process	GO:0036003	positive regulation of transcription from RNA polymerase II promoter in response to stress	12	0	0.02272	12	1	nhr-49	nhr-202-nhr-49|nhr-49-WBGene00004804|nhr-49-WBGene00022703|nhr-49-WBGene00010251|nhr-49-nhr-49|nhr-49-WBGene00002147|nhr-49-WBGene00077712|nhr-49-WBGene00008852|farl-11-WBGene00013355|dcar-1-WBGene00010251|dcar-1-WBGene00001251|dcar-1-WBGene00077712
GO biological process	GO:0060625	regulation of protein deneddylation	7	0	0.01948	6	1	F22B8.7	F22B8.7-WBGene00012039|F22B8.7-WBGene00011195|F22B8.7-WBGene00018330|F22B8.7-WBGene00008296|F22B8.7-WBGene00013606|F22B8.7-WBGene00011304

GO biological process	GO:0048729	tissue morphogenesis	51	0	0.01737	39	1	farl-11	C05C8.7-WBGene00005024|farl-11-WBGene00000395|farl-11-WBGene00000390|farl-11-WBGene00000961|farl-11-WBGene00001330|farl-11-WBGene00001165|farl-11-WBGene00006876|farl-11-WBGene00003258|farl-11-WBGene00014153|farl-11-WBGene00005020|farl-11-WBGene00005021|farl-11-WBGene00005022|farl-11-WBGene00000168|farl-11-WBGene00005023|farl-11-WBGene00005024|farl-11-WBGene00005025|farl-11-WBGene00005026|farl-11-WBGene00006868|farl-11-WBGene00006869|farl-11-WBGene00001687|farl-11-WBGene00016354|farl-11-WBGene00006437|farl-11-WBGene00019030|farl-11-WBGene00010631|farl-11-WBGene00000199|farl-11-WBGene00002181|farl-11-WBGene00000200|farl-11-WBGene00006957|farl-11-WBGene00000204|farl-11-WBGene00000205|farl-11-WBGene00000203|farl-11-WBGene00013355|farl-11-WBGene00006961|farl-11-WBGene00021170|farl-11-WBGene00003241|farl-11-WBGene00004749|farl-11-WBGene00007561|farl-11-WBGene00001333|farl-11-WBGene00001526
